# Genome sequencing and analysis of *Alcaligenes faecalis* subsp. phenolicus MB207

**DOI:** 10.1038/s41598-018-21919-4

**Published:** 2018-02-26

**Authors:** Zarrin Basharat, Azra Yasmin, Tongtong He, Yigang Tong

**Affiliations:** 1 Microbiology & Biotechnology Research Lab, Department of Environmental Sciences, Fatima Jinnah Women University, Rawalpindi, 46000 Pakistan; 2 State Key Laboratory of Pathogen and Biosecurity, Beijing Institute of Microbiology and Epidemiology, Beijing, 100071 China

## Abstract

Bacteria within the genus *Alcaligenes*, exhibit diverse properties but remain largely unexplored at genome scale. To shed light on the genome structure, heterogeneity and traits of *Alcaligenes* species, the genome of a tannery effluent isolated *Alcaligenes faecalis* subsp. phenolicus MB207 was sequenced and assembled. The genome was compared to the whole genome sequences of genus *Alcaligenes* present in the National Centre for Biotechnology Information database. Core, pan and species specific gene sequences i.e. singletons were identified. Members of this genus did not portray exceptional genetic heterogeneity or conservation and out of 5,166 protein coding genes from pooled genome dataset, 2429 (47.01%) contributed to the core, 1193 (23.09%) to singletons and 1544 (29.88%) to accessory genome. Secondary metabolite forming apparatus, antibiotic production and resistance was also profiled. *Alcaligenes faecalis* subsp. phenolicus MB207 genome consisted of a copious amount of bioremediation genes i.e. metal tolerance and xenobiotic degrading genes. This study marks this strain as a prospective eco-friendly bacterium with numerous benefits for the environment related research. Availability of the whole genome sequence heralds an opportunity for researchers to explore enzymes and apparatus for sustainable environmental clean-up as well as important compounds/substance production.

## Introduction

*Alcaligenes* specie strains exist in soil, water, and environment, as well as in association with humans. The bacteria of this genus are usually non-pathogenic but occasional opportunistic infections could occur in humans. Bacterial species belonging to the genus *Alcaligenes* have demonstrated versatile pollutant bioremediation capability, including phenols^1,2^, phenanthrene^3^, polyaromatic hydrocarbon^4,5^, pesticides^6,7^ and azo dye degradation^8^. *Alcaligenes faecalis* has been reported to convert the most toxic form of arsenic, arsenite to its less dangerous form, arsenate. Tolerance to heavy metals has been reported as well^9,10^. Nanoparticle production^11^, nematicidal^12^ and biocontrol activity^13^ has been reported in addition to production of chemicals^14^, detergent^15^, gum^16^, and bioplastics^17^. Despite such high applicability of *Alcaligenes* species in major spheres of research and prospective benefits in industry, agricultural and environmental domain, it remains underrepresented and understudied at whole genome level. No major comparative analysis or pan-genomic analysis has been published related to this bacterium till to date.

Here, we report the genome features of an *Alcaligenes* specie as well as comparison of its pan-genome and microsatellite i.e. simple sequence repeat (SSR)/ compound microsatellite (cSSR) profile with other *Alcaligenes* specie genomes. *Alcaligenes faecalis* subsp. phenolicus MB207 was isolated in 2010 from the effluent of a tannery in Multan, located in the southern zone of the province of Punjab in Pakistan and its genome was sequenced as a part of the on-going project on understanding and applying micro-remediation for environmental clean-up. Our group is further working on the various molecular aspects of this bacterium both *in vitro* and *in silico*, to further shed light on the mechanisms beneath its bioremediation capability.

## Results

### Overview of the sequenced genome

**Figure 1:**
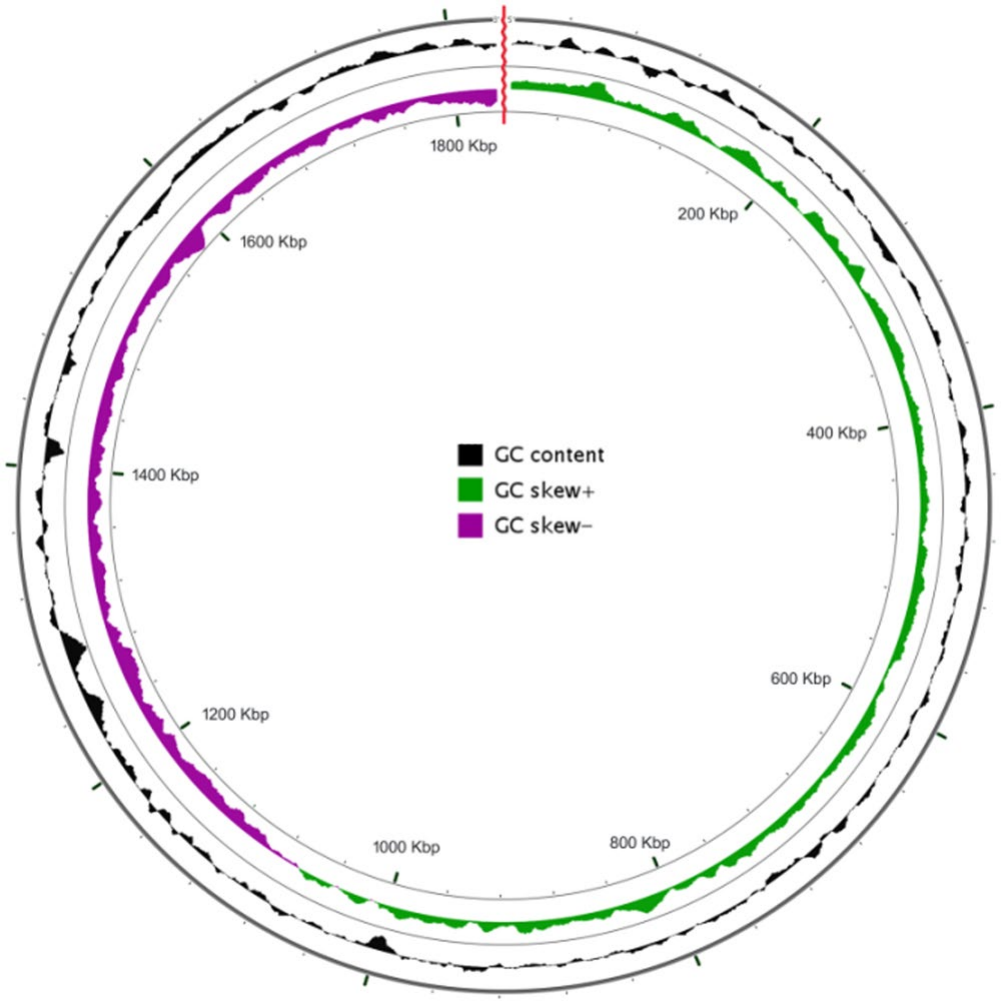
Graphical map showing the assembled genome sequence of *Alcaligenes faecalis* subsp. phenolicus MB207. Replication origin of length 918 nucleotides i.e. region 10243–11160 is shown by a red divider ring with 3′ leader to the left and 5′ trailer at its right side.

Total length of the genome was 4,156,248 bp with a GC content of 56.4%. Genome assembly through IDBA-UD approach resulted in 9 scaffolds. The sequence reads have been deposited in the NCBI SRA database and allocated the accession number: SRR5809679. OrthoANI value of 96.0 was obtained after comparison with the type strain *Alcaligenes faecalis* subsp. phenolicus DSM 16503. PGAP annotation revealed that the genome comprised of 3,812 genes, out of which 3,749 were coding DNA sequences and 63 were RNA genes. A total of 63 RNA genes were detected, including 5 S rRNA, 16 s rRNA and 23S rRNA copies. 53 tRNAs coding for all 20 amino acids and 4 ncRNAs were predicted. Pseudogenes with ambiguities like frameshift error, internal stop as well as incomplete pseudo gene sequences were predicted along with origin of replication (Fig. 1).

Incomplete prophage regions were predicted and apart from cell proliferation, chemotaxis, type I, II, VI secretion system, chaperones for cold shock, colicin V, lipase, patatin, siderophore production and antibiotic resistance proteins, biphenyl mineralization, phenol degradation, metal resistance, azo dye and Ibuprofen degradation proteins were found. Key features of some of the resistance genes are shown in Table 1. Genome sequence of *Alcaligenes faecalis* subsp. phenolicus MB207 harbours multiple xenobiotic degrading enzymes, enabling it to tolerate and thrive in the presence of anthropogenic, toxic compounds. All these genes were most probably encoded by chromosome as plasmid could not be detected. The presence of a repertoire of genes with bioremediation capability provides a genomic foundation for micropollutant tolerance ability of this versatile bacterium.

**Table 1:** Antibiotic, metal and pollutant resistance gene and their translated producted features from the PGAP annotated *Alcaligenes faecalis* subsp. phenolicus MB207.

Serial no.	Gene name	Nucleotide position	Gene length	Protein molecular weight	Isoelectric point	Basic residues	Acidic residues
1.	Arsenical resistance protein ArsH	225909..226625	716	26999.90	6.5327	32	30
2.	Multidrug transporter subunit MdtC (resitant to novobiocin and deoxycholate)	351991..355095	3104	112426.50	7.7024	94	86
3.	Copper resistance protein CopZ	589513..590028	515	19203.20	6.2496	17	16
4.	Bcr/CflA family drug resistance efflux transporter	820699..821934	1235	42612.95	9.4199	26	15
5.	Arsenic resistance protein	1469622..1470530	908	32769.09	10.6979	20	9
6.	Fusaric acid resistance protein	1382547..1384538	1991	72089.74	9.9998	72	40
7.	Copper resistance protein CopC	1369230..1370852	1622	58417.92	7.0504	46	35
8.	Antibiotic resistance protein	949611..950324	713	25195.75	9.2276	19	11

*Alcaligenes* spp have been used for bioplastic production with fatty acid supplementation^18^ and in presence of sugar beet/cane-molasses as sugar and urea as carbon source^19^. Our genome also encompassed enzymes for polyhydroxyalkanoate (PHA) synthesis, repression and depolymerization. Our strain carries genes for PHA (linear polyesters) production, which is usually a product of lipid and sugar fermentation by bacteria in nature. The gene is in immediate vicinity of acetyl-CoA enzyme which aids condensation of two acetyl-CoA molecules to acetoacetyl-CoA which is further reduced to the monomer hydroxybutyryl-CoA, the building block of PHA^20^. Intracellular stockpiling of PHA is usually carried out in the nutrient limiting and excess carbon setting, where PHA is hoarded inside the cell as energy-reserve granules. The biodegradable PHA polymer resembles petrochemical based polymer, which is unfortunately non-degradable. This heralds good news for eco-friendly bioplastic synthesis and reduce plastic related pollution. The presence of a PHA repressor protein shows a fine-tuning of the mechanism through negative regulation. Negative feedback loop could support homeostasis during environmental flux. A PHA depolymerizing enzyme occurrence indicates its possible role in plastic degradation and in turn aiding environment clean-up.

**Figure 2:**
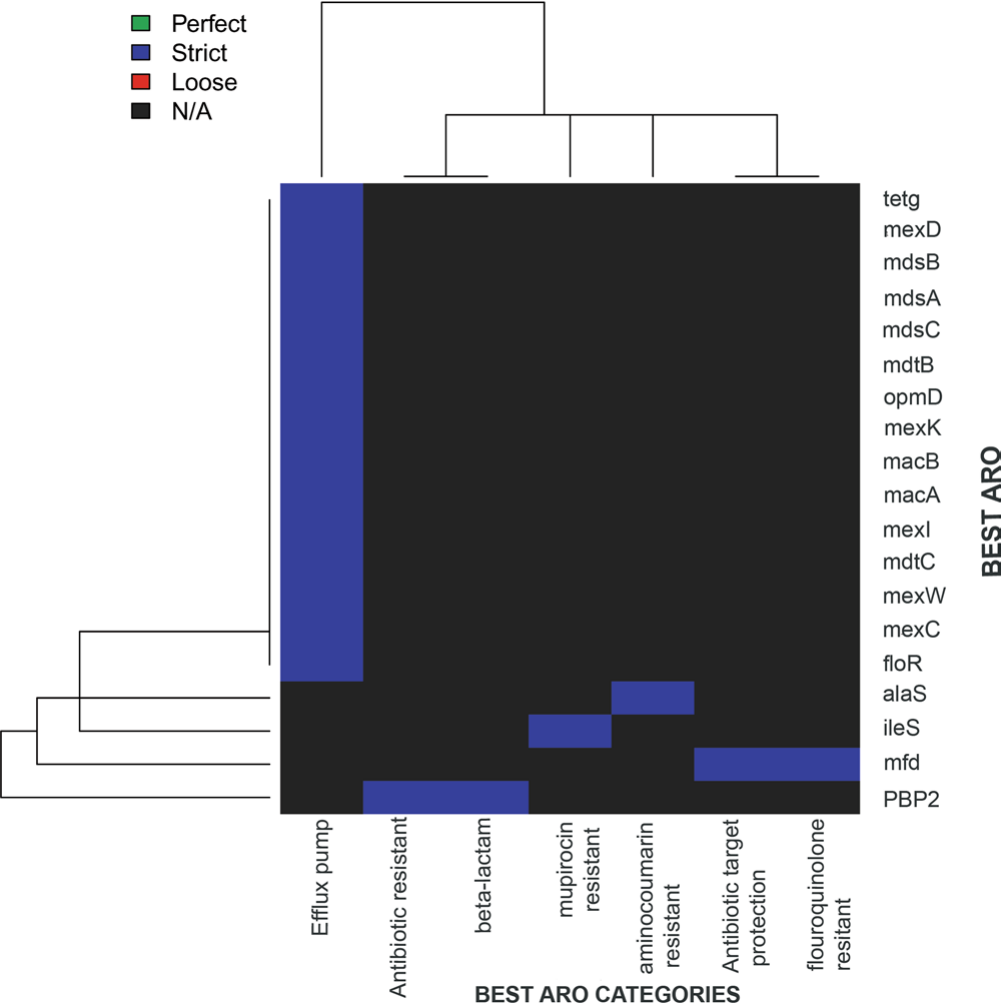
Antibiotic Resistance Ontology (ARO) based heatmap diagram showing similarity of strict matches (blue colour) for antibiotic resistance genes of other bacterial species and our strain MB207. A similarity to 19 genes conferring antibiotic resistance was observed.

Antibiotic resistance is a pressing issue with natural history as well as human use. Many bacteria are avid antibiotic producers and resist them as well, for survival^21^. Genes from these are transferred horizontally to the antibiotic susceptible strains, resulting in acquiring resistant genes. In *Alcaligenes faecalis* subsp. phenolicus MB207, we catalogued the antibiotic resistance genes to understand the tannery polluted environmental reservoir of such genes. Only one sequence i.e. Undecaprenyl pyrophosphate phosphatase, involved in the sequestration of undecaprenyl pyrophosphate had a similarity cut-off above threshold and showed resistance to bacitracin. Other sequences had a lower cut-off value but high BLAST similarity and depicted resistance to tetracycline, chloramphenicol, fluoroquinolone, aminoglycoside, macrolide, trimethoprimlincosamide, macrolide, streptogramin_b, tigecycline, beta-lactam, carbenicillin, penicillin, erythromycin, glycylcycline, roxithromycin, kasugamycin, streptomycin, acriflavine, puromycin and t_chloride. Antibiotic resistance was also observed for mupirocin and anticoumarin via CARD (Fig. 2).

### Pollutant tolerance

*Alcaligenes faecalis* subsp. phenolicus MB207 demonstrated tolerance to micropollutants including heavy metals^22^ (up to 250 µg/ml for nickel, cadmium, copper, zinc, lead and chromium in LB medium; pH:7; Temperature: 37 °C) and pharmaceutical Ibuprofen (our unpublished data). Genes for metal tolerance were searched and multiple copies of bacterioferritin, porins, ABC transporters, ATPases etc were found which are key regulators of metal transport in and out of the cell, involved in metal detoxification and survival in metal-stressed environment. Protein families for sensing and regulation for specific metals like arsenic and copper were also present.

*Alcaligenes faecalis* subsp. phenolicus MB207 has also shown azo dye (sulphonated mono and di-azo dye methyl orange and Congo red respectively) degradation capability^23^ (Supplementary Fig. 1). A degradation percentage of 64.18, 68.97 and 77.97 was achieved in LB medium (pH:7; Temperature:37 °C; Concentration: 100 µg/ml) for Congo red after 24, 48 and 72 hours respectively while a degradation percentage of 19.48, 36.8, and 40 was achieved for Methyl orange in LB medium after 24, 48 and 72 hours respectively. This is comparatively low as compared to other bacterial strains isolated from polluted environment, such as *Pseudomonas*^24^ with degradation as high as 97% achieved in 12 hours in similar conditions. A decolourization of 96 and 87% was achieved in 12 hrs for Methyl orange and double the quantity of Congo red (i.e. 200 µg/ml) respectively, at 30 °C for *Shewanella xiamenensis* BC01^25^. However, no data for degradation of both these sulphonated azo dyes is available for comparison with other *Alcaligenes faecalis* species. Although degradation percentage seemed low but it exhibited a remarkable capability of growth with both these dyes as sole carbon source. Genes responsible for the degradation of these dyes previously reported in literature such as azoreductase and peroxidase (GenBank accession: OQV32989.1 and OQV32923.1) were present in the genome sequence. BLAST hits showed closely related (up to 99% similar) NAD(P)H quinone dehydrogenase and azoreductase sequences in other *Alcaligenes* sp. genomes. A similarity of only 48% with azoreductase structure (of *Pseudomonas puti*da, PDB ID: 4C14) in the Protein Data Bank was obtained. For peroxidase, high similarity was obtained for sequences in related genomes and only a 53% similarity was obtained with human peroxiredoxin structure in the Protein Data Bank. (PDB ID: 5B6M). There is a strong need for the prediction/solving of structures for these proteins.

### Pan-genomic analysis

**Figure 3:**
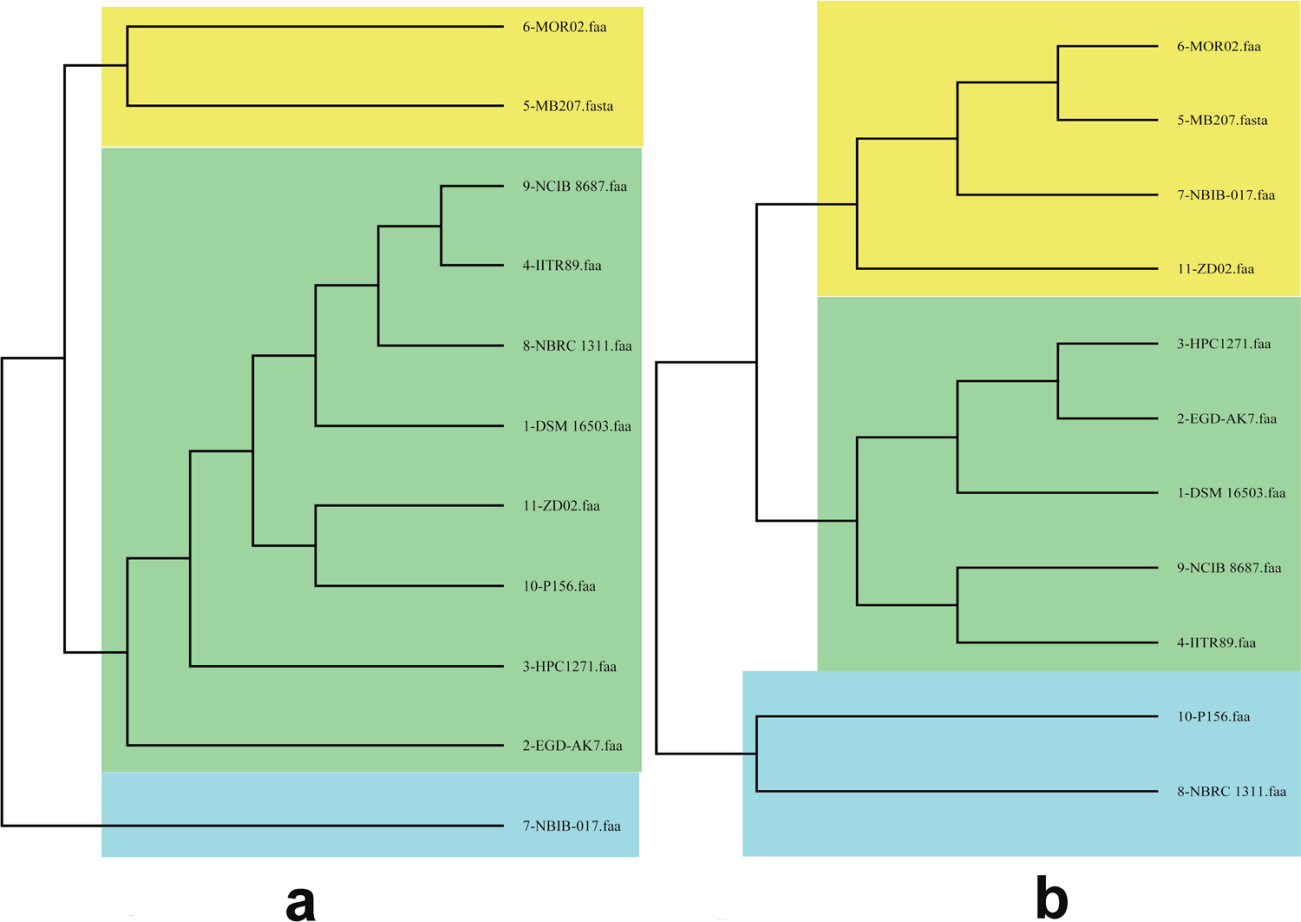
Phylogenetic analysis of the genus *Alcaligenes* based on (**a**) core and (**b**) pan-genome similarity. Clusters have been coloured separately to distinguish groups. Serial numbers referring to the genome names are same as the ones allocated in Table 2.

**Figure 4:**
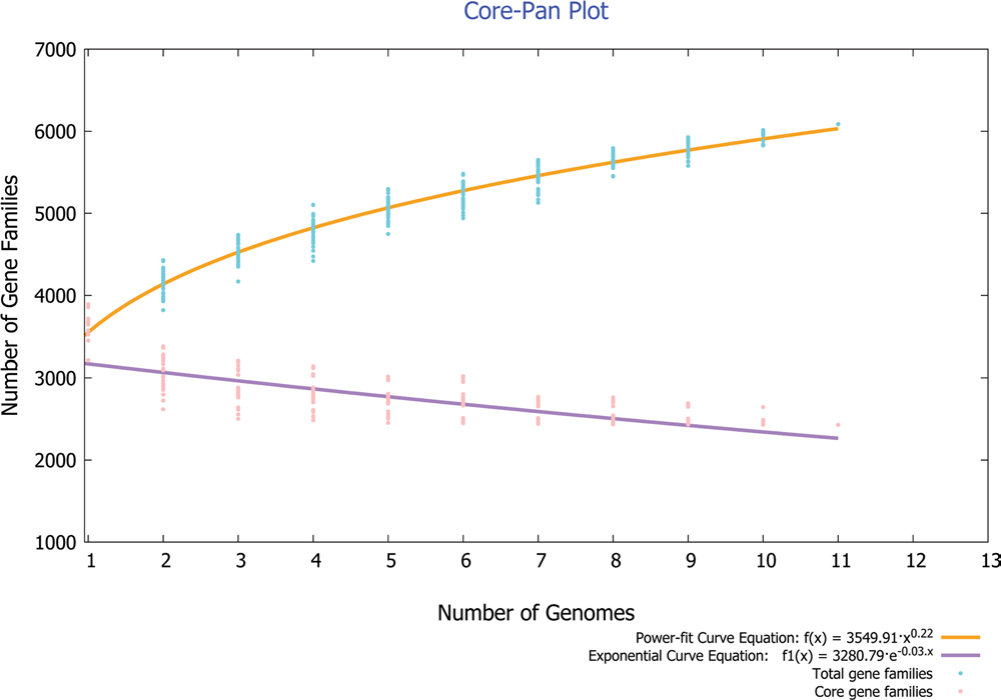
The pan and core genome plot of studied genomes. Total gene families are shown by blue colour while pink colour represents core gene families.

This type of analysis when applied to the sub-groups of organisms helps differentiate the serovars and pathovars, through niche and virulence-specific gene segregation. Distinct ecological and pathogenic traits could thus, be sieved out through this approach. Pan-genomic information been used as an aid to therapeutic design in bacteria^26,27^ as well as for heterogeneity study^28^. A total of eleven genomes of genus *Alcaligenes* were subjected to pan-genomic analysis (Table 2). The cluster map was significantly altered for the pan and core genome centred phylogeny for the studied bacterial species, apart from our bacterium *Alcaligenes faecalis* subsp. phenolicus MB207 and *Alcaligenes faecalis* M0R2, which remained grouped together (Fig. 3). BPGA tool also determined pan and core genome curve (Fig. 4) and its extrapolation through power law, to assess closing or openness of the pan-genome. The expected size of the pan-genome was calculated as 6087 while size estimated was 6032. The parameter ‘b’ was calculated to be 0.221095 and the pan-genome curve showed a plateau formation, indicating that pan-genome of this genus is yet open but may be closed soon. We have previously postulated that a pan-genome should be permanently open in bacteria due to natural evolution and horizontal gene transfer and we believe it should be the same case in genus *Alcaligenes*.

**Table 2:** Characteristics of genome sequences of the genus *Alcaligenes* present in the NCBI database to date and used for pan-genome analysis with our strain MB207. Number of core genes calculated for all these species amounted to 2,429.

Serial No.	Bioproject ID	Bacterial specie	No. of contigs	Genome size (Mbp)	DNA G + C content (%)	No. of accessory genes	No. of unique genes	No. of exclusively absent genes
1	PRJNA185539	*Alcaligenes faecalis* subsp. phenolicus DSM 16503(Type)	27	4.24831	56.40	1041	201	46
2	PRJNA215169	*Alcaligenes* sp. EGD-AK7	70	4.28184	56.62	1324	104	2
3	PRJNA175407	*Alcaligenes* sp. HPC1271	78	4.27093	56.58	1035	73	217
4	PRJNA307081	*Alcaligenes faecalis* subsp. phenolicus IITR89	23	3.77406	57.60	665	117	61
5	PRJNA360554	*Alcaligenes faecalis* subsp. phenolicus MB207	9	4.15625	56.4	1168	120	1
6	PRJNA258399	*Alcaligenes faecalis* MOR02	23	4.40271	56.35	1203	260	3
7	PRJNA300936	*Alcaligenes faecalis* NBIB-017	17	4.16548	56.40	1129	87	4
8	PRJDB275	*Alcaligenes faecalis* subsp. faecalis NBRC 13111(Type)	29	4.03369	56.65	1055	88	5
9	PRJNA86069	*Alcaligenes faecalis* NCIB 8687	186	3.89962	57.17	773	248	6
10	PRJNA312705	*Alcaligenes faecalis* P56	31	4.02769	56.70	987	108	14
11	PRJNA276624	*Alcaligenes faecalis* ZD02	10	4.26552	56.82	1113	138	3

**Figure 5:**
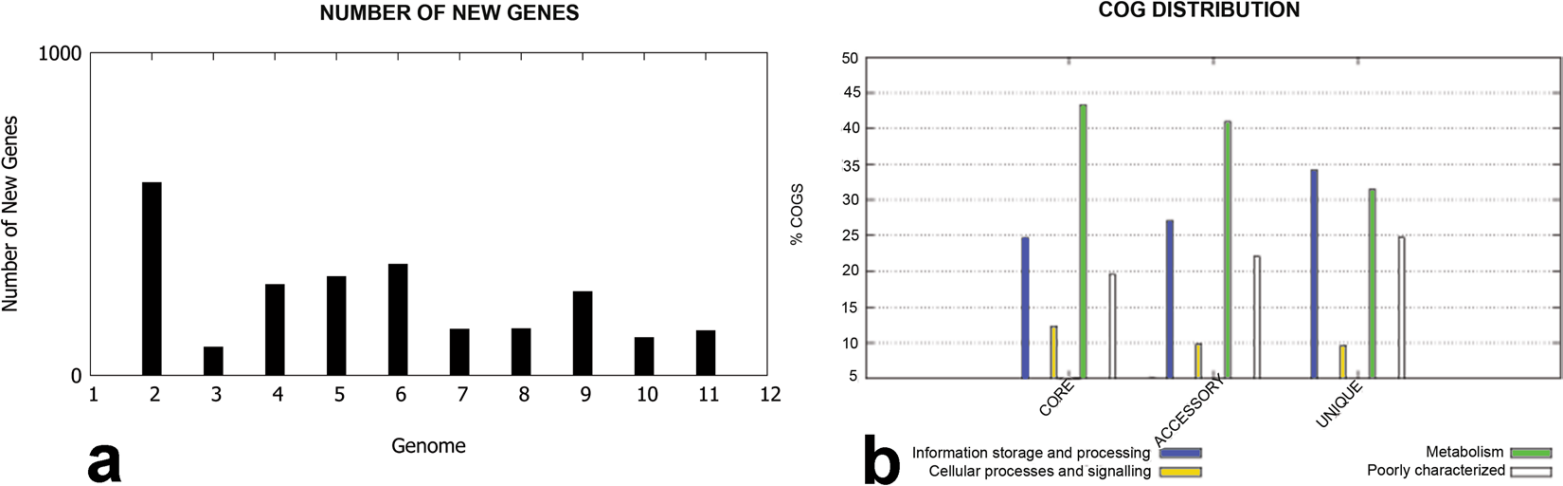
(**a**) Number of new genes contributing to pan-genome for upon sequential addition of each genome. (**b**) COG distribution of the genes making up core, accessory and unique portion of the studied genomes.

The highest number of new genes which contributed to the pan-genome were observed for *Alcaligenes* sp. EGD-AK7 (Fig. 5a). Bulk of the core genome of this genus was composed of genes having metabolic related functionality (Fig. 5b) as previously observed for the genus *Serratia*^29^, whereas hypothetical/poorly characterized genes contrived most of the unique or specie specific genome.

### Secondary metabolite producing gene cluster analysis

Secondary metabolites serve as a rich source of bioactive compounds with pharmaceutical and other important properties. The compendium of genes encoding for important metabolites was detected through blast search against genes with similar architecture and composition. The genus has been underexplored for secondary metabolite genes which usually exist in clusters. Our genome was compared against gene cluster database (with information for ~3000 clusters) and predicted to encompass six such metabolite producing clusters.

The first cluster (Location: 169171–180010 nt) consisting of eleven genes, encoded butyrolactone. Homologous cluster were mined from *Alcaligenes* sp. EGD-AK7 and *Alcaligenes* sp. HPC1271 with a 28 percent similarity. Components include the major biosynthetic gene (Afsa) with A-factor biosynthesis hotdog domain, vital to streptomycin production and resistance. Major facilitator transporter was located upstream as well as downstream of biosynthetic gene. Siderophore receptor, damage inducible protein and MarR family transcriptional regulator coding genes were located upstream while downstream region comprised of TetR transcritional regulator which regulates processes like antibiotic production, resistance, efflux pump expression and osmotic stress response regulation. Second cluster (Location: 458937–469329 nt) codes for ectoine product. Out of thirteen genes, product synthesizing ones include diaminobutyrate-2-oxoglutarate-transaminase and L-ectoine synthase. Ectoine hydrolase and transporters were located downstream while metalloprotease, diaminobutyrate acetyltransferase, transcriptional regulator of class MarR and EamA family transporter were present upstream. More than 20 genes were mined upstream and downstream of terpene synthesis gene cluster with a total length of 21730 nucleotides. Ribose-5-phosphate isomerase, arsenate reductase, putrescine transporter components, sythases for squalene, phosphate starvation induced proteins, 3-deoxy-D-manno-octulosonic acid kinase, alanine racemase, alanine transaminase, homoserine dehydrogenase, periredoxin, zeta-carotene desaturase and DNA repair protein made up this cluster. Resorcinol producing cluster (Location: 920642–962552 nt) had homologs in four *Alcaligenes* species with similarity ranging from 24 to 17 percent. ABC transporters, dehydrogenases, TetR and LysR family transcriptional regulators, eneterobactin esterase, quercetin 2,3-dioxygenase, amidohydrolase and decarboxylating condensing enzymes coding for type III polyketide synthases, made up this cluster.

**Figure 6:**
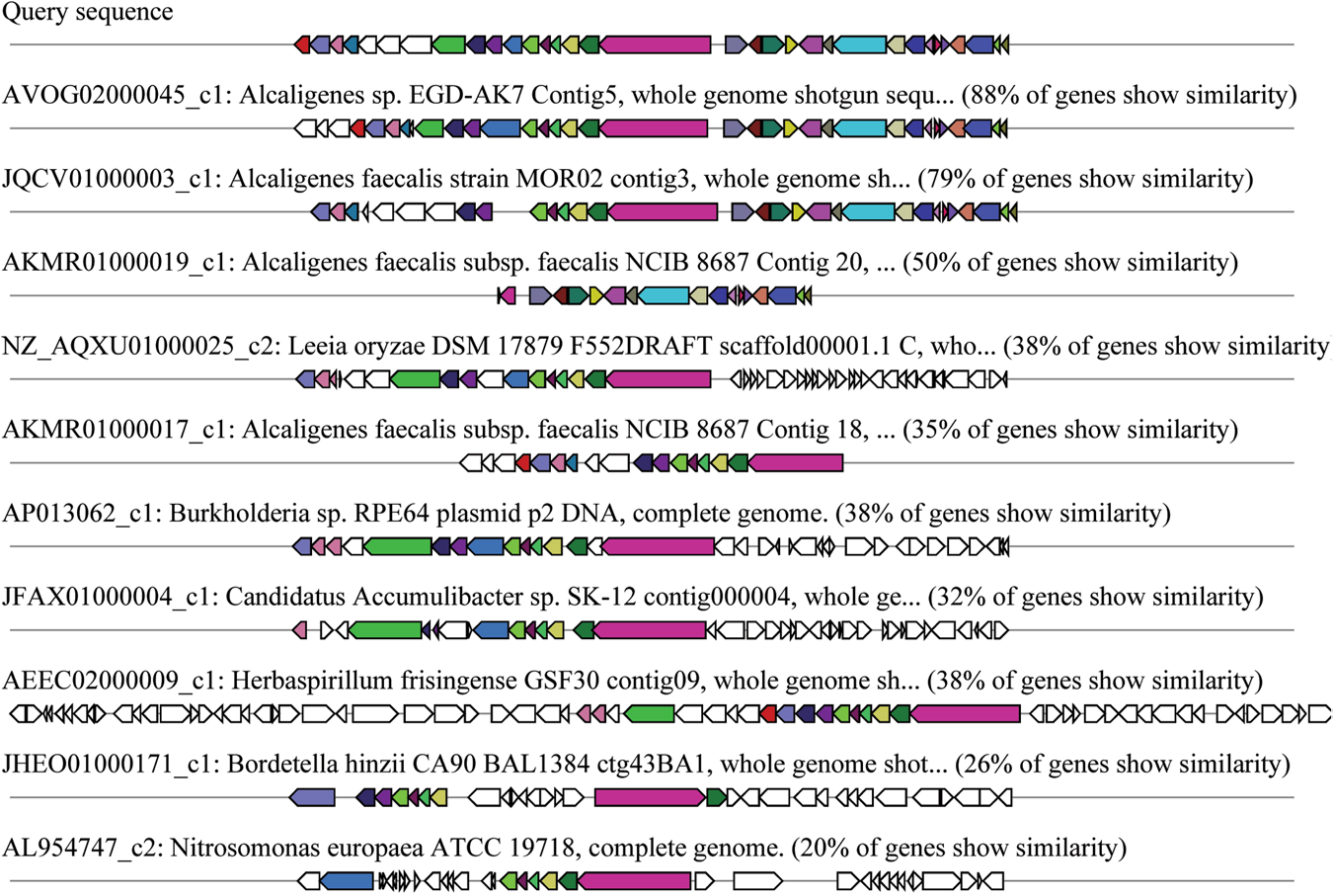
Top ten polyketide synthase gene clusters homologous to *Alcaligenes faecalis* subsp. phenolicus MB207. Putative biosynthetic genes presented in red, transport-related genes in blue and regulation-related genes in green colour.

All of the genes for type I non-ribosomal peptide synthesis cluster (Location: 597271–644863 nt) genes showed some similarity with *Alcaligenes* sp. EGD-AK7 and *Alcaligenes faecalis* subsp. faecalis NCIB 8687 non-ribosomal peptide synthesis cluster (Fig. 6). This cluster constituted biotin metabolizing enzyme 8-amino-7-oxononanoate synthase, permeases, hypothetical proteins, polysaccharide deacetylase, glycosyl transferase, ABC transporter machinery, spore coat forming protein and capsular biosynthesis protein.

### SSR and cSSR analysis

*Alcaligenes* sp. dataset consisted of 30,610 SSRs and 455 cSSRs ranging from a minimum of 2501 SSRs to a maximum of 3224. cSSRs ranged from 31 to 65 (Table 3). SSR density ranged from ~661 to 775 (Mean = 2782.73, S.D. = 204.07), while cSSR density ranged from 7.68 to 15.64 (Mean = 41.36, S.D. = 10.72). Strain MB207 had the highest number of SSR and cSSRs among the studied genomes. Strong correlation between SSR and cSSR density (R^2^ = 0.73, P < 0.01) was observed. This was almost similar to the previously analysed *Lactobacillus* genomes but dissimilar to the *Escherichia coli* genomes. This is in accordance with the hypothesis that the correlation of SSR and cSSR density is specie specific and dependent upon recombination of SSR motifs instead of the replication phenomenon^28^. Correlation between GC content and cSSR density (R^2^ = 0.13, P = 0.27) as well as genome size and cSSR density (R^2^ = 0.17, P = 0.19) was weak and non-significant contradictory to the results from other analyzed bacterial genomes^28,30^. Increment in cSSR formation is usually observed upon increase in maximum allowed distance between two adjoining SSRs. Number of cSSRs in all *Alcaligenes* specie genomes also increased with increased in d_MAX_ values (Table 3).

**Table 3:** SSR and cSSR analysis of *Alcaligenes* specie genomes. SSR density, cSSR density and number of overlapping cSSRs at both d_MAX_ = 10 and d_MAX_ = 50 are shown.

Serial No.	Bacterial specie	No. of SSRs	No. of cSSRs (d_MAX_ = 10)	No. of cSSRs (d_MAX_ = 50)	SSR density (d_MAX_ = 10)	cSSR density (d_MAX_ = 10)	No. of overlapping compound cSSRs (d_MAX_ = 10)	No. of overlapping compound cSSRs (d_MAX_ = 50)
1	*Alcaligenes faecalis* subsp. phenolicus DSM 16503(Type)	2782	42	97	654.89	9.89	10	34
2	*Alcaligenes* sp. EGD-AK7	2840	41	110	663.21	9.57	12	29
3	*Alcaligenes* sp. HPC1271	2863	39	109	670.33	9.13	7	23
4	*Alcaligenes faecalis* subsp. phenolicus IITR89	2501	31	93	662.69	8.21	11	33
5	*Alcaligenes faecalis* subsp. phenolicus MB207	3224	65	115	775.74	15.64	20	32
6	*Alcaligenes faecalis* MOR02	2935	50	118	666.59	11.36	16	31
7	*Alcaligenes faecalis* NBIB-017	2751	33	98	660.50	7.92	11	29
8	*Alcaligenes faecalis* subsp. faecalis NBRC 13111(Type)	2657	31	99	658.65	7.68	8	23
9	*Alcaligenes faecalis* NCIB 8687	2546	33	104	652.82	8.46	9	35
10	*Alcaligenes faecalis* P56	2623	37	96	651.19	9.19	12	27
11	*Alcaligenes faecalis* ZD02	2888	53	117	676.98	12.42	8	26

To determine the organization and imperfection in SSR motif arrangement, giving rise to cSSRs in *Alcaligenes* genomes, we explored the complexity and structural make-up of cSSRs. cSSR coupled motifs (e.g. TTAAGT-CTTGTT) were unique i.e. distinct for each *Alcaligenes* specie and most probably arose by defective duplication.

Motif duplication i.e. similar motifs on both ends of spacer sequence existed once in *Alcaligenes faecalis* subsp. phenolicus DSM 16503 and *Alcaligenes* sp. EGD-AK7, at both d_MAX_ = 10 and d_MAX_ = 50. In *Alcaligenes faecalis* NBIB-017, *Alcaligenes faecalis* subsp. phenolicus IITR89, *Alcaligenes* sp. HPC1271, *Alcaligenes faecalis* MOR02, *Alcaligenes faecalis* strain NCIB 8687, no duplication was observed at d_MAX_ = 10 but at d_MAX_ = 50, adjacent duplications of 1, 1, 2, 2 and 2 motifs were mined respectively. Presence of similar duplicated motifs advocates that these *Alcaligenes* species have analogous array of motif duplication in their genomes. For *Alcaligenes faecalis* subsp. phenolicus MB207, 3 duplications at d_MAX_ = 10 and 2 duplications at d_MAX_ = 50 were observed. *Alcaligenes faecalis* NBIB-017 showed 4 and 3 pair of duplications at d_MAX_ = 10 and d_MAX_ = 50 respectively. In *Alcaligenes faecalis* ZD02, presence of 1 and 2 duplicated motifs were detected at d_MAX_ = 10 and d_MAX_ = 50 respectively. *Alcaligenes faecalis* strain P156 did not show any duplication at either d_MAX_ = 10 or d_MAX_ = 50.

Moreover, a cSSR is either categorized as perfect (e.g. [(TG)_n_(TA)_n_]) or overlapping (intersection of final base of the precedent motif with first base of the next motif (e.g [(ATT)_n_(TG)_n_]) (Kumar *et al*., 2014). Overlapping cSSR motifs were present in all *Alcaligenes* genomes (Table 2). Inspection of cSSR complexity indicated that cSSR assembly was very complex and intricate in the *Alcaligenes* genome sequences. A complexity of up to ‘32-microsatellite’ cSSRs was reached in our sequenced genome specie at d_MAX_ = 50, which is even greater than that of eukaryote ‘24-microsatellite’ complexity^31^.

## Discussion

*Alcaligenes faecalis* subsp. phenolicus is a gram-negative rod-shaped bacterium. It has the unique ability to utilize phenol as a sole carbon source^1^. Here, we have sequenced and reported the characteristics of *Alcaligenes faecalis* subsp. phenolicus MB207. Sequencing provided a glimpse into its micropollutant tolerance capability and gene apparatus responsible for these properties was identified and being investigated further, both *in vitro* and *in silico*. Our isolate had micropollutant resistance, azo dye and ibuprofen degradation properties. This bacterium has ample genes for metal sensing and transport which enables it to create metal homeostasis system that helps it survive/thrive in polluted environment. Further analysis like varied expression profiling and proteome alteration under metal stress could provide better understanding regarding metal homeostasis in the bacterium. Genome sequencing of *Alcaligenes faecalis* subsp. phenolicus MB207 is an important milestone in understanding its remediation and eco-friendly properties. A lot of antibiotic resistance genes were mined and since antibiotic resistance system impacts metal homeostasis and vice versa, it would be interesting to explore this facet too.

We also elucidated the bioplastic forming and depolymerizing apparatus in addition to gene clusters responsible for butyrolactone, ectoine, resorcinol, terpene, nrps and pks. In silico inspection in *Alcaligenes* specie genomes revealed a wealth of SSRs and cSSRs. The most complex structured cSSR was detected in our sequenced genome. It is demonstrated that previously uncorrelated genome data can be utilized for mining of new biological information by means of available softwares, databases and high-performance computation. Democratization of genome sequencing has made bacterial genomics a mature and easy approach for researchers from interdisciplinary fields like environment, evolution and scientists working in the biomedical disciplines. Genomic data stored in repositories is available to public for comparison with their own datasets which has made the studies concurrently deeper and diffused, leading to interesting results and conclusions. A striking example is the concept of pangenome, introduced originally with pathogenic strains and now widely studied for non-pathogenic species/genus of interest. We have touched upon this analysis but for full scale comparison with other bacteria of similar sizes and to make solid conclusions, similar scale approaches need to be undertaken. The software and parameters need to be standardized for quality assessment. Secretion system protein clusters did not show significant alignment with a particular genus or specie although each protein of the system resembled a similar protein of different specie but on the whole, system level conservation was not observed, although interspecies similarity was high.

Tandem repeats of nucleotide motifs (sized 1–6 bp) are called SSRs and give rise to cSSRs upon joining. They are known to exist in all genomes and their importance ranges from use as molecular markers to studying genome evolution. cSSRs have an alleged role in the expression of gene regulation and functional dictation of proteins in numerous species^30,32^. Hence, it is important to study their distribution, enrichment and polymorphism in the genomes of interest. Correlation between SSR and cSSR density of our isolate was almost similar to the previously analysed *Lactobacillus* genomes but dissimilar to the *Escherichia coli* genomes. This is in accordance with the hypothesis put forward by us^28^ that the correlation of SSR and cSSR density is specie specific and dependent upon recombination of SSR motifs instead of the replication phenomenon. Since the correlation of GC content with cSSR density and genome size with cSSR density was weak and non-significant. This did not comply with the previous analyses on bacterial genomes^28,30^. cSSR coupled motif structure was consistent with *Escherichia coli* and *Lactobacillus* cSSR motifs, with distinct bases^30^ unlike eukaryotic cSSRs, that have similar motifs in ~90% cases^31^. Occurrence of ‘32-microsatellite’ cSSR complexity divulges from previously analysed *Escherichia coli* and *Lactobacillus* genome study, which did not cross the maximum complexity of more than ‘5-microsatellite’ cSSRs upon dMAX increment to 50. Complexity of prokaryotic cSSR does not seem to be dependent upon genome size as genome size of eukaryotes is colossal as compared to genome size of bacteria. This is also consistent with previous study that complexity seems to be depends on SSR abundance as it might augment the frequency of joining SSRs into cSSRs by chance^28^. Our analysis is computational and only an intelligent guess due to lack of absolute certainty in the GC content and genome sizes of the genomes used in the study i.e. unfinished/draft genome sequences. The actual impact needs to be proven experimentally.

## Methods

### Genome sequencing, assembly and annotation

DNA extraction was done with high pure PCR template preparation kit (Roche, Switzerland). Whole-genome sequencing was performed using the MiSeq PE300 sequencer with 2 × 300 bp pair-end library. A total of 794,028 reads (55× coverage of the genome) were generated, cleaned and quality filtered using Trimmomatic^33^. Reads were then corrected for errors through String graph assembler which utilizes a k-mer centric algorithm^34^. *De novo* assembly was attempted through IDBA-UD algorithm centred on *de bruijn* graph approach^35^. Genome annotation was carried out using the NCBI ‘Prokaryotic genome annotation pipeline’. Genomic context was visualized through GView^36^. Prophage regions were identified using PHAST^37^ and antibiotic resistance was profiled through BLAST module of ARDB^38^. Resistance Gene Identifier (RGI) listed at CARD site (https://card.mcmaster.ca/analyze/rgi), was used for mapping of resistome featuring homology and SNP model with strict criteria.

### Specie demarcation and pan-genomic analysis

OrthoANI^39^ scheme was used for specie demarcation using whole genome sequence data. This type of orthologous average nucleotide identity (ANI) calculation between genomes is valid for differentiation at the species scale for microorganisms. A value of 95% and above indicates that the queried bacterium belongs to the same species as that of the reference.

BPGA (Bacterial Pan-genome Analysis tool) was used for estimation of core, pan and specie specific genome analysis^40^. The thresholds of the score and E-value used for BLAST were greater than 50 and less than 1e^−8^, respectively. Annotated genomes were taken as a seed substance for the construction of a pan-genome. *Alcaligenes faecalis* genomes present in the NCBI database (till the accomplishment of this study i.e. March 2017) were subjected to a pair wise homology search through BLAST. Orthologs were calculated for all possible genome pairs. In case of a partial or incomplete gene sequence, the reciprocity cannot be marked clearly due to small length. Even with a similarity of 100%, gene cannot be captured in accessory or core genome data pool and labelled as a singleton. To circumvent this problem, a length of 50% or more was considered for gene reciprocity. Initial clustering was done through Usearch algorithm and output processed into pan, core and accessory gene distribution of the genus *Alcaligenes*. The empirical power law equation f(x) = a.x^b and exponential equation f1(x) = c.e^(d.x) were used for extrapolation of the pan and core genome curves respectively. Exclusive presence and absence of genes/families was determined to infer specie-specific gene families. Upon addition of each new genome in the analysis pipeline, 20 random permutations of genomes were carried out to circumvent any bias. Evolutionary analysis based on concatenated gene alignments and binary (presence/absence) pan-matrix was conducted with Neighbour joining approach. COG distribution, KEGG pathway analyses and phylogeny based on core and pan proteome was then attempted.

### Secondary metabolite analysis

antiSMASH^41^ was used for secondary metabolite gene cluster detection as well as detailed comparison to related clusters in other microorganisms. It is based on hidden Markov model profiling of genes associated with important metabolite production of all known broad chemical classes. The boundary of the gene clusters is estimated via various greedily chosen cut-off values, specified per gene cluster type and genes represented in specified colours referring to certain functionality.

### SSR and cSSR analysis

SSR and cSSR information was extracted using the software IMEx^42^ in batch, using the parameters: Include flanking regions: 10 bp, Type of repeat: imperfect; Repeat Size: all; Minimum Repeat Number: Mono: 12, Di: 6, Tri: 4, Tetra: 3, Penta: 3, Hexa: 3, Imperfection limit/repeat unit: Mono: 1, Di: 1, Tri: 1, Tetra: 2, Penta: 2, Hexa: 3, Percent imperfection in repeat tract: 10%, Maximum distance allowed between any two adjacent SSRs forming a cSSR (i.e. d_MAX_ in bp): 10 with complete standardization^28^. The obtained results were then compared to microsatellites in previously studied prokaryotic species i.e. *Escherichia coli*^30^ and *Lactobacillus*^28^. Linear regression (R^2^) was calculated using IBM SPSS v22, to evaluate the impact of GC content and genome size on the SSR and cSSR composition as well as correlation among SSR density (number of SSR/Mb) and cSSR density (number of cSSR/Mb). A P-value of <0.05 was considered as significant.

### Nucleotide sequence accession numbers

The accession numbers of the sequences of *Alcaligenes faecalis* subsp. phenolicus MB207 determined in this study can be found in GenBank (http://www.ncbi.nlm.nih.gov) under the accession no. MTBI01000001-MTBI01000009.

## Electronic supplementary material

Supplementary Information 
