# Supplementary material for: Genome sequencing and analysis of Alcaligenes faecalis subsp. phenolicus MB207
Source: Sci Rep. 2018 Feb 26;8:3616. doi: 10.1038/s41598-018-21919-4 (PMC5827749; doi:10.1038/s41598-018-21919-4)
Supplement: Supplementary file 1 — Supplementary Information [file 41598_2018_21919_MOESM1_ESM.pdf]

Genome sequencing and analysis of *Alcaligenes faecalis* subsp. phenolicus MB207

Zarrin Basharat[a], Azra Yasmin[a*], Tongtong He[b], Yigang Tong[b]

[a] Microbiology & Biotechnology Research Lab, Department of Environmental Sciences, Fatima Jinnah Women University, Rawalpindi 46000, Pakistan.

[b] State Key Laboratory of Pathogen and Biosecurity, Beijing Institute of Microbiology and Epidemiology, Beijing 100071, China.

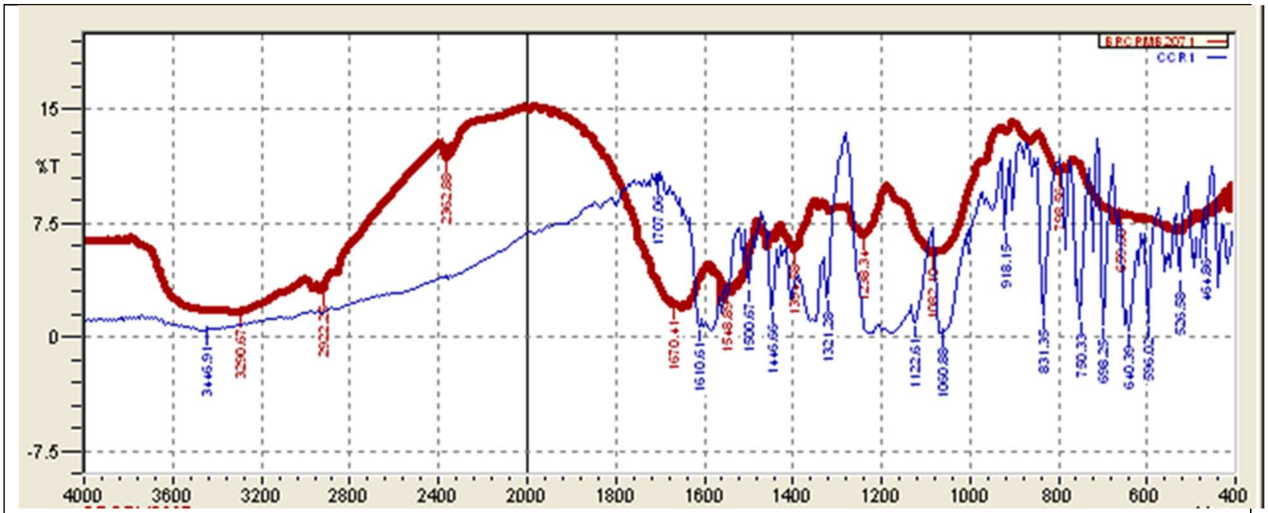

a

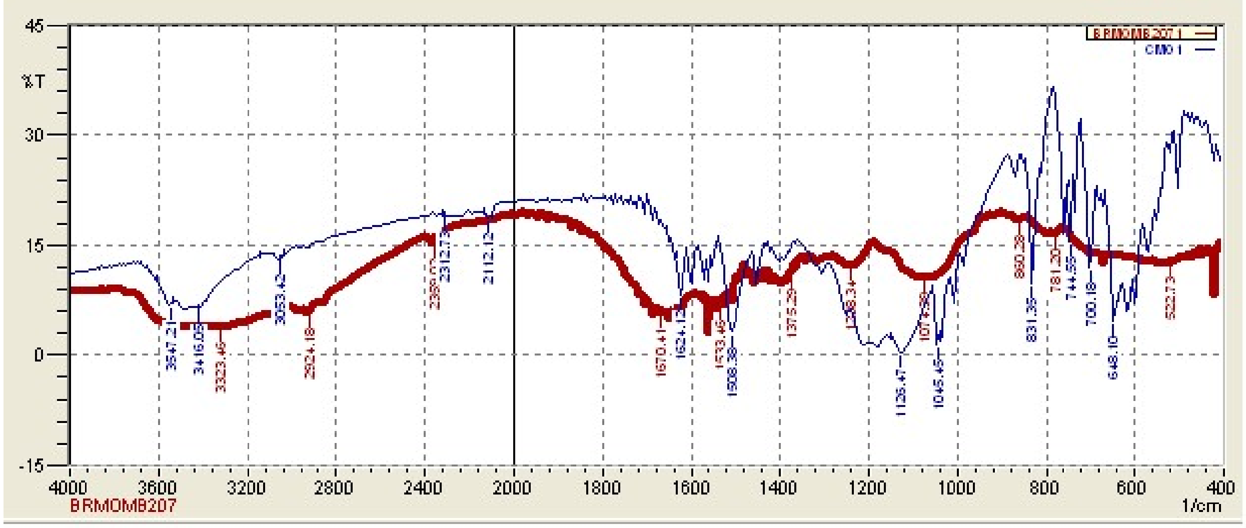

b

Supplementary Fig. 1. FTIR spectrum of control i.e. (a) Methyl orange dye vs degraded Methyl orange and (b) Congo Red vs degraded Congo red by *Alcaligenes* sp. MB207 in LB-broth.

FTIR analysis of Congo red control showed specific peaks in the fingerprint region 1650 cm^{-1} (for amide functional group), 1300 cm^{-1} N=N symmetric stretching vibration of azide group, 1310 cm^{-1} (ring structure vibration), around 1400 cm^{-1} (napthalene ring), 1505-1550 cm^{-1} (C=C vibration in the naphthalene ring), 1530 cm^{-1} (N=N stretching vibration) , 1080 cm^{-1} (C-N stretching vibration of amine),around 990 cm^{-1} (S=O stretching vibration), 1110 cm^{-1} (Ring vibration) including C-S and symmetric SO_3 stretching vibration, 800 cm^{-1} (C-C skeletal vibration), 660 cm^{-1} (C-S stretching and vibration). FTIR spectrum of degraded Congo red product by *Alcaligenes* sp. MB207 showed

characteristic peaks at 3900 cm^{-1} (O-H stretching vibration), 2930 cm^{-1} (C-H stretching vibration), 2940 cm^{-1} (N-H stretching vibration), 2400 cm^{-1} (N-H stretching vibration), 1600 cm^{-1} (C=C stretching vibration probably at –diazene position), 1450cm^{-1} (asymmetrical C-H deformation vibration), 1500 cm^{-1} (N-H bending vibrations),1198 cm^{-1} (C-H skeletal vibration), 989 cm^{-1} (C-C skeleton vibration), 900 cm^{-1} (N-H out-of-plane bending vibrations), 950 cm^{-1}(C-C stretching vibration), 880 cm^{-1}(C-N stretching vibration of amine), 790 cm^{-1} (C-H out-of-plane deformation vibrations of meta-di-substituted aromatic compounds) and 500 cm^{-1} (C-C skeleton vibration).

FTIR analysis of Methyl orange control showed specific peaks at 3100 cm^{-1} (asymmetric C-H stretching vibration), 3060 cm^{-1} (=C-H stretching vibration), 2112 cm^{-1} (C-C and CN stretching vibrations), 1700 cm^{-1} (C=C stretching vibration), 1624 cm^{-1} (C=C stretching vibration), 1610 cm^{-1} (C=C stretching vibration), 1585 cm^{-1} (C=C stretching vibration), 1500 cm^{-1} (C-H symmetric deformation vibration), 1385 cm^{-1} (symmetric C-H deformation vibration), 1186 cm^{-1} (C-H skeletal vibration), 1050 cm^{-1} ($C-H_3$ rocking vibration),1010 cm^{-1} (C-C skeleton vibration), 990 cm^{-1} (C-C skeleton vibration), 845 cm^{-1} (C-H vibration), 800 cm^{-1}(aromatic C-H out-of-plane deformation vibrations), 720 cm^{-1} (CH_3 rocking vibration), 655 cm^{-1} (C-H wagging vibration), 642 cm^{-1} (Ring deformation), 595 cm^{-1} (C-C skeletal vibrations ,510 cm^{-1}(C-C skeletal vibration), 495 cm^{-1} (skeletal vibration), 410 cm^{-1} (skeletal vibration), 445 cm^{-1} (skeletal vibration). Degraded product showed peaks at around 2900 cm^{-1} (C-H stretching vibration), 3100 cm^{-1} (N-H stretching vibration-associated to =C), 2400 cm^{-1} (asymmetric N-H stretching vibration), 1700 cm^{-1} (C=C stretching vibration), 1600 cm^{-1} (C-H vibration), 1506 cm^{-1} (N-H deformation vibrations), 1200 cm^{-1} (C-H skeletal vibration), 1050cm^{-1} (C-H symmetrical deformation vibration), 831 cm^{-1} (C-H out-of-plane deformation vibration), 770 cm^{-1} (N-H out-of-plane bending vibrations).
